# Determinants of Medication Adherence to Antihypertensive Medications among a Chinese Population Using Morisky Medication Adherence Scale

**DOI:** 10.1371/journal.pone.0062775

**Published:** 2013-04-25

**Authors:** Gabrielle K. Y. Lee, Harry H. X. Wang, Kirin Q. L. Liu, Yu Cheung, Donald E. Morisky, Martin C. S. Wong

**Affiliations:** 1 School of Public Health and Primary Care, The Chinese University of Hong Kong, Hong Kong SAR, China; 2 New Territories East Cluster, Hospital Authority, Hong Kong SAR, China; 3 Department of Community Health Sciences, UCLA Fielding School of Public Health, United States of America; University of Ottawa, Canada

## Abstract

**Background and Objectives:**

Poor adherence to medications is one of the major public health challenges. Only one-third of the population reported successful control of blood pressure, mostly caused by poor drug adherence. However, there are relatively few reports studying the adherence levels and their associated factors among Chinese patients. This study aimed to study the adherence profiles and the factors associated with antihypertensive drug adherence among Chinese patients.

**Methods:**

A cross-sectional study was conducted in an outpatient clinic located in the New Territories Region of Hong Kong. Adult patients who were currently taking at least one antihypertensive drug were invited to complete a self-administered questionnaire, consisting of basic socio-demographic profile, self-perceived health status, and self-reported medication adherence. The outcome measure was the Morisky Medication Adherence Scale (MMAS-8). Good adherence was defined as MMAS scores greater than 6 points (out of a total score of 8 points).

**Results:**

From 1114 patients, 725 (65.1%) had good adherence to antihypertensive agents. Binary logistic regression analysis was conducted. Younger age, shorter duration of antihypertensive agents used, job status being employed, and poor or very poor self-perceived health status were negatively associated with drug adherence.

**Conclusion:**

This study reported a high proportion of poor medication adherence among hypertensive subjects. Patients with factors associated with poor adherence should be more closely monitored to optimize their drug taking behavior.

## Introduction

Poor adherence to medications is a major public health challenge. Adherence to medication has been defined as the extent to which patients’ behaviors coincide with health care providers’ recommendations for health and medical advice [1]. It can be defined as the extent to which a patient’s behavior, with respect to taking medication, corresponds with agreed recommendations from a healthcare provider [2]. Hypertension is one of the most common non-communicable diseases globally. More than 26% of the adult populations worldwide have been diagnosed as having hypertension, and the prevalence of hypertension increases with age [3]. Globally, it is also one of the major causes of premature death, and 7.1 million of people die from hypertension related diseases annually and the problem is still growing [4].

Successful control of blood pressure is of paramount importance in the reduction of morbidity and mortality rates [5], and many studies have demonstrated the impact of antihypertensive agents on improving clinical outcomes 6–8. However, the effectiveness of antihypertensive agents must be achieved by optimal adherence to prescribed medications according to healthcare providers’ instructions [9].

Although the control of blood pressure has improved considerably, poor adherence with medication treatment remains a major problem among hypertensive patients, and has been identified as one of the main causes of failure in achieving blood pressure control [10]. Only 29% of hypertensive patients in the United Stated achieved good control, and even worse rates have been reported in Canada and European countries [11]. It is estimated that the overall adherence rates of medications were approximately 50% [12]. Among hypertensive patients who have poor blood pressure control; poor drug adherence is one of the causes, and accounts for increasingly significant and substantial public health burden. Of all avoidable hospital admissions in the United States, 33 to 69 percent are due to poor medication adherence, with expenses of approximately $100 billion a year [13], [14]. Poor adherence has been attributed to unnecessary over-prescription of drugs, substantial worsening of diseases, avoidable increases in hospital admission rates, longer hospital stays, leading to a significant medical burden [15], [16]. It is a crucial public health agenda to improve adherence with antihypertensive medications by improvements of medication-taking behavior.

Barriers to drug adherence consist of multiple factors that include complex medication regimens, dosing frequency, behavioral factors and side effects of treatment [16]. The most typical barriers to drug adherence are under the patient’s control, including patient’s knowledge and attitudes towards medications. Therefore, attention to these barriers is a necessary and important step to improve adherence [16]. Various methods have been developed to measure medication adherence, and they can be grouped into three categories: subjective (self-report), direct (serum or urine drug-level), and indirect (pharmacy database records, pharmacy refill rates, or pill counts) [10], [17]. Each method has its strengths and drawbacks; and subjective and indirect methods are more frequently used in adherence-related studies. Self-reported measures are a relatively simple and inexpensive method, and it could also include information on social, situational and behavioral factors that affect adherence [10], [17].

Antihypertensive drug adherence has been extensively evaluated in many developed countries in the West using different methods of medication adherence measurement [18]–[20]; however there is a scarcity of studies evaluating the adherence levels of antihypertensive pharmacotherapies among Chinese population.

This study aims to assess the drug adherence profiles of Chinese patients prescribed antihypertensive agents, and to examine the factors associated with antihypertensive drug adherence among Chinese patients. Self-reported survey as a tool to measure drug adherence was used in this study to supplement the findings of previous research reports. As this study evaluated additional factors including education level, and self-perceived health status which have not been explored among Chinese patients in previous studies [21]–[23], the findings of this study could provide more comprehensive information for physicians and health care policy makers with targets to improve medication adherence.

## Methods

### Ethics Statement

The study was approved by Joint Chinese University of Hong Kong - New Territories East Cluster Clinical Research Ethics Committee (CREC) of Hong Kong. The study was conducted within the researcher’s country of residence. All participants provided their written informed consent to participate in this study.

### Setting and Subjects

This study was conducted in one of the out-patient clinics in the New Territories Region from 01 February 2012 to 30 April 2012. This clinic is a public, primary care clinic run by trained family physicians. There are around 500 patient visits daily, excluding evening sessions. All adult patients aged 18 years or older; who are taking at least one long-term antihypertensive drug; and are able to communicate and understand Cantonese were invited to participate. Each potential participant was verbally asked about the nature of their chronic medication and verified by their hand-held records to ascertain study eligibility.

### Study Design and Protocol

This study is a cross-sectional survey and the data were collected primarily by self-administered questionnaires. All eligible patients were invited to complete a self-administered questionnaire consisting of basic socio-demographic profile, self-perceived health status, and self-reported medication adherence. University students who have attended a standardized workshop on study protocol and questionnaire administration consecutively screened for eligible participants and invited them to complete a survey after obtaining patient consent. For illiterate patients, the student researchers read the question items word-by-word as exactly printed on the questionnaire. Each participant had their blood pressure and body mass index (BMI) measured after survey completion. The standardized workshops aim to enhance inter-rater reliability.

### Demographic and Clinical Information of the Study Participants

Participants’ age, gender, level of education, marital status, number of antihypertensive drug taking, duration of taking antihypertensive drugs, educational levels, occupation, marital status and self-perceived health status were obtained through a self-reported survey. The BMI was measured by a calibrated stadiometer and weight scale for the study participants on light clothing without shoes. BMI was further classified by the following groups: underweight (<18 kg/m^2^); normal (18–23 kg/m^2^); overweight or obesity (>23 kg/m^2^). Self-perceived health status consists of the following categories: excellent and good, not good and not bad, poor and very poor. It was evaluated by a questionnaire item designed based on the Health Belief Model [24] using a 5-point Likert scale.

### Assessment of Medication Adherence

Self-reported medication adherence was measured by the eight-item Morisky Medication Adherence Scale (MMAS-8). The MMAS-8 has been proven reliable (alpha = 0.83) for assessment of adherence in patients with hypertension, and is significantly associated with blood pressure control [25]–[27]. Using a cut-off of 6, its sensitivity or identifying low vs. higher adherers was estimated to be 93%. and the specificity was 53% [26]. The MMAS-8 has been demonstrated to have good concurrent and predictive validity and might function as a screening tool in outpatient settings with different patient groups [26]. MMAS-8 scores can range from zero to eight in integers (see Appendix S1). The precise scoring criteria can be obtained from the developer/owner. The advantages of this instrument over other methods of measurement include its simplicity, quick administration and low-cost. A Chinese version of the MMAS-8 [28] was used and adapted specifically for antihypertensive agents in this study. The survey was in Cantonese, tailored-made for use in the Hong Kong population.

### Outcomes Variables and Covariates

The outcome variable in this study is the measure of drug adherence as assessed by self-reported Morisky scores [26], [27], [29], transformed into a dichotomous variable with a score greater than 6 considered as “good adherence”. The independent variables that were controlled for include age, gender, the total number of antihypertensive drugs used irrespective of medication class, duration of antihypertensive drug use, educational levels, marital status, occupation, monthly household income, self-perceived health status, systolic blood pressure and diastolic blood pressure. Blood pressure was measured using standardized methodology based on the 2007 guidelines for the management of arterial hypertension [30]. All patients had their BP measured twice, each separated 10 minutes apart. The average of two BP values was used in the analysis.

### Statistical Analysis

The PASW Statistics version 18.0 (SPSS) was used for all data analysis. In the univariate analysis, all variables listed above were consecutively tested for significant associations with “good adherence” as the outcome variable. Chi-square tests and Student’s t-tests were used for categorical and continuous variables, respectively. All variables were unconditionally entered into a binary logistic regression model for adjustment. The absence of multicollinearity was ascertained before the regression modeling. As part of quality control, each data entered into the computer were double checked for their accuracy by random assessment of the survey responses and the data entered. All p values ≤0.05 were regarded as statistically significant.

## Results

A total of 1,154 consecutive patients completed and returned the questionnaires. Of those, 24 (2.1%) reported not taking antihypertensive agents and 7 (0.6%) were missing, and theywere excluded from the analysis. There were 1,114 completed surveys included in the analysis. The basic characteristics of these patients were shown in Table 1. Among the eligible patients, the majority of patients (60.1%) reported taking one antihypertensive agent and the mean number of antihypertensive agents used was 1.6 (SD 1.0). The mean year of antihypertensive used was 7.7 years (SD 6.9). The mean systolic blood pressure was 130.6 mmHg (±16.9) and the mean diastolic blood pressure was 74.7 mmHg (±9.3). The mean score of the MMAS-8 was 6.7 (±1.4), ranging from 0 to 8. A total of 65.1% of patients obtained a score of greater than 6 and their adherence was categorized as “good”, while 32.6% of patients obtained a score of 6 or below and adherence levels were classified as “poor”.

**Table 1:** Patient characteristics.

		Total study population (N = 1,114)	
		N	%
**Gender**	Male	464	41.7
	Female	648	58.3
**Age**	Mean (SD)		65.7 (11.1)
	<60	341	31.0
	60–65	242	22.0
	66–69	110	10.1
	≥70	406	36.9
**No. of antihypertensive drugs used**	Mean (SD)		1.6 (1.0)
	1	662	60.1
	2	325	29.5
	≥3	114	10.4
**Duration of antihypertensive drugs used (year)**	Mean (SD)		7.7 (6.9)
	<5 years	531	47.9
	5–10 years	336	30.3
	>10 years	241	21.8
**Body Mass Index** *	Underweight	26	3.2
	Normal Weight	210	25.9
	Overweight or Obesity	583	70.9
**Marital status**	Married	838	76.1
	Single/Divorced/Widowed/Cohabited	263	23.9
**Education level**	Primary or below	597	54.0
	Secondary	440	39.8
	Tertiary or above	68	6.2
**Occupation**	Employed	282	25.8
	Unemployed/Retired	531	48.5
	Housewife	281	25.7
**Monthly Household income**	0–10000	501	57.0
	100001–20000	222	25.3
	20001–30000	89	10.1
	30001+	67	7.6
**Current Health Status**	Excellent or Good	269	24.5
	Not Good and Not Bad	645	58.9
	Poor or Very Poor	182	16.6
**Systolic blood pressure**		1,081	130.6/16.9 (Mean/SD)
**Diastolic blood pressure**		1,080	74.7/9.3 (Mean/SD)

* Body Mass Index is calculated as weight in kg/(height in meters)^2^.

The characteristics of patients who had good adherence to antihypertensive agents and patients who had poor adherence were mostly similar (Table 2). When compared with patients who had good adherence to antihypertensive agents, more patients who had poor adherence to antihypertensive agents were younger (39.2% vs 27.0% were aged <60), had shorter duration of antihypertensive agents used (45.2% vs 36.7% were first users of antihypertensive agents within the past 5 years), more were employed (31.9% vs 22.2%), and reported a self-perceived health status of “poor or very poor” (18.9% vs 15.2%).

**Table 2:** Patient characteristics according to antihypertensive drug adherence.

		Good adherence		Poor adherence		*p*
		n	%	n	%	
**Total Study Population**		725	65.1	363	32.6	
**Age**	Mean (SD)	718	67.4 (10.9)	355	64.8 (11.2)	<0.001
	<60	194	27	139	39.2	
	60–65	149	20.8	85	23.9	
	66–69	80	11.1	27	7.6	
	≥70	295	41.4	104	29.3	
**Gender**		723		363		0.073
	Male	314	43.3	137	37.7	
	Female	409	56.4	226	62.3	
**No. of antihypertensive used**		716	1.5 (0.8)	359	1.6 (1.1)	0.813
	1	427	59.6	221	61.6	
	2	215	30	104	29	
	≥3	74	10.3	34	9.5	
**Duration of antihypertensive used (year)**		722	8.0 (6.8)	361	7.6 (6.9)	0.006
	<5 years	265	36.7	163	45.2	
	5–10 years	283	39.2	137	37.7	
	>10 years	174	24.1	61	16.8	
**Body Mass Index (kg/m^2^)** *		526		265		0.989
	Underweight	17	3.2	9	3.4	
	Normal Weight	133	25.3	70	26.4	
	Overweight or Obesity	376	71.5	186	70.2	
**Education level**		719		360		0.246
	Primary or below	402	55.9	183	50.8	
	Secondary	272	37.8	155	43.1	
	Tertiary or above	45	6.3	22	6.1	
**Marital status**		716		360		0.961
	Married	544	76.0	274	76.1	
	Single/Divorced/Widowed/Cohabited	172	24.0	86	23.9	
**Occupation**		715		357		<0.001
	Employed	159	22.2	114	31.9	
	Unemployed/retired	386	54	137	38.4	
	Housewife	170	23.8	106	29.7	
**Monthly Household income ($HK)**		571		291		0.472
	0–10000	335	58.7	155	53.3	
	100001–20000	138	24.2	80	27.5	
	20001–30000	54	9.5	33	11.3	
	30000+	44	7.7	23	7.9	
**Current Health Status**		719		355		0.069
	Excellent or Good	189	26.3	73	20.6	
	Not Good and not Bad	421	58.6	215	60.6	
	Poor or Very Poor	109	15.2	67	18.9	
**Systolic Blood Pressure**	Mean (SD)	724	130.8 (16.36)	358	130.5 (17.11)	0.704
**Diastolic Blood Pressure**	Mean (SD)	724	73.5 (9.04)	357	75.3 (9.43)	0.853

* Body Mass Index is calculated as weight in kg/(height in metres)^2^.

All variables, which showed significant correlations with the adherence score, were entered into a multivariate logistic regression model. After adjustment of potential confounders, good adherence to antihypertensive agents was more common among those with advanced age, patients who have used antihypertensive agents for more than ten years, and those who were unemployed or retired (Table 3). As a sensitivity analysis, unemployed patients were separated from the retired and the identical logistic regression model was repeated. The associated factors found were similar to that in the original regression analysis (data not shown). In addition, patients who reported a self-perceived health status of “poor or very poor” were more likely to report poor adherence to antihypertensive agents (Table 3).

**Table 3:** Factors associated with good adherence to antihypertensive drugs.

	No.	Adjusted OR (95% C.I.)	*p*
**Age (years)**	1,042	1.016 (1.001–1.032)	0.037
**Duration of taking antihypertensive drugs (years)**			0.022
<5 years	492	1.000	0.065
5–10 years	319	1.334 (0.982–1.810)	0.011
>10 years	231	1.598 (1.115–2.291)	
**Occupation**			
Employed (Full-time/Part-time)	266	1.000	0.041
Unemployed/Retired	513	1.496 (1.017–2.200)	0.041
Housewife	263	1.012 (0.696–1.473)	0.949
**Self-perceived health status**			
Excellent/Good	256	1.000	1.126
Not good and Not bad	619	0.789 (0.570–1.092)	0.153
Poor/Very poor	167	0.647 (0.422–0.991)	0.045

The following variables were entered into a binary logistic regression model: age, gender, number of antihypertensive agents used, duration of antihypertensive agents used, body mass index, educational level, marital status, occupation, monthly household income, self-perceived health status, systolic blood pressure and diastolic blood pressure.

## Discussion

The purpose of this study was to study the adherence profiles of Chinese patients prescribed antihypertensive agents and the factors associated with drug adherence using a self-administered questionnaire. The overall rate of good adherence to antihypertensive agents (65.1%) was not as high as a previous study among Chinese population (85.5%) [10], and was slightly lower than the adherence rate of 71.6% among patients in Urban health Clinic Setting using the MMAS-8 in the U.S [31]. Other studies in western population reported rates ranging from 53% to 91% [32], [33]. The rate of adherence may differ by many factors, including types of population, study design, method of measurement, to quote a few. It has been reported that cultural factors might influence antihypertensive adherence which could explain different levels of adherence among different populations. These include cultural health perception of hypertension, health perceptions of Western medications, self-care behavior and social support [34].

Several studies have also evaluated the factors associated with antihypertensive drug adherence [35]–[37]. Age, gender, the number of antihypertensive agents, and socioeconomic status were found to be associated with drug adherence. One of the studies conducted in a Chinese population [38] has found that that many of the factors associated with antihypertensive drug adherence among Chinese patients were similar to those identified by studies conducted in Western populations. Among Chinese patients, there were previous studies which utilized Medication Possession Ratio (MPR) as a measure of adherence from retrospective dataset analysis [30], [38]–[42]. These reports were different from this study which prospectively evaluated medication adherence using a self-reported instrument. We are of the view that both types of studies could be interpreted in different perspectives to inform the current situation of medication non-adherence in Chinese patients.

The association between age and drug adherence is complex and the conclusion is currently mixed in the literature. In this study, patients with older age were found to be more adherent than patients with younger age. This finding was compatible with the majority of studies that advanced age was reported to be associated with good adherence to antihypertensive agents [10], [17], [31], [43], [44]. A possible explanation is the greater number of comorbidities among elderly patients, who might perceive themselves as sicker and hence adhere better to antihypertensive prescriptions [34]. But conversely, some researchers reported the opposite correlation where poor adherence was associated with increasing age [36], [45]. Other variables like individual’s cognitive level, physical mobility, and self-care abilities may affect the relationship between age and adherence.

In this study, patients with longer duration of antihypertensive agents used (over 10 years) reported better adherence than patients with shorter duration (5 years or less). This finding was consistent with a previous study using the MMAS-8 where poor antihypertensive drug adherence was more commonly found among those first diagnosed with hypertension for less than 10 years [31]. One explanation for this could be that patients having longer duration of taking antihypertensive agents could have gained more experience with hypertension, established a better physician-patient relationship and had greater faith on physicians’ advice. In addition, they might become more knowledgeable about their own health condition and the appropriate management of disease control [46].Turning to occupation, patients who were unemployed or retired were more likely to be compliant. Few studies have reported the relationship between occupation and adherence. Some studies have shown that unemployed patients tended to have poorer adherence [47]; and the lack of health care coverage is a hidden issue that has emerged as important. Health care systems and practices of different countries may affect the result [48]. It is possible that the positive association between unemployed or retired statuses and good adherence to antihypertensive agents was mediated by having low-cost subsidy, and that medical service was relatively accessible in Hong Kong.

In addition, patients who reported a self-perceived health status of “poor or very poor” were less likely to be adherent to antihypertensive agents when compared with those who reported a self-perceived health status of “excellent or good”. Patients’ perception and attitudes towards health have been recognized as one of the barriers to drug adherence [16]. It is therefore possible that patients reported a self-perceived health status of “poor or very poor” have a relatively poorer self-control and negative beliefs about hypertension, limiting their adherence to antihypertensive medication regimes.

To our knowledge, this is the first study that evaluated the adherence profile of antihypertensive agents among Chinese patients using the measuring tool of the MMAS-8. Self-reported survey as a tool to measure drug adherence used in this study supplemented the findings of previous studies. As additional factors including education level, and self-perceived health status where previous studies have not explored in Chinese population were evaluated in this study, the findings of this study provide clear implications to physicians and health care policy makers. It is obvious that patients with the identified associated factors should receive more intensive monitoring for drug adherence. Effective strategies to improve drug adherence can be achieved by reinforcement of regular monitoring of blood pressure at home, educational programmes involving family member, consultations by community pharmacists, and sending reminders of clinical appointments and regular drug taking [48].

Several limitations of our study should be addressed here. First, our sample was taken in a single out-patient clinic in the New Territories region, and patients’ characteristics may differ from other districts, hence limiting its representativeness and it may not be generalizable to other health care settings. Secondly, we have excluded those hypertensive patients who were unable to communicate and understand Cantoneseand this could over-estimate the adherence level by including patients with higher educational levels than the general public. Also, research methodologies involving self-reported measures depend largely on individuals’ memory, and recall bias may exist. In additional, many other factors such as barriers to medication taking, patients’ knowledge and attitudes towards long-term drug use which could influence adherence were unable to be controlled. Lastly, the absence of association between the Morisky scores and the blood pressure levels is unexpected. One explanation is that we have not excluded patients having white-coat hypertension despite the standard methodology to measure BP. Further studies are needed to evaluate the relationship between this scale and BP levels among Chinese hypertensive patients.

### Conclusions

In conclusion, some factors associated with antihypertensive drug adherence among Chinese patients using the MMAS-8 were novel findings as compared with studies conducted in Chinese patients using Medication possession ratio (MPR) [30], [38]–[42] and other studies conducted in Western population. Since studies on drug adherence to antihypertensive agents among Chinese population is scarce, further investigation is required to evaluate the applicability of MMAS as a tool of drug adherence measurement among Chinese patients. The factors identified in this study denote those individuals at risk for poor antihypertensive drug adherence, and future research studies should identify the reasons for their non-adherence. More drug adherence-enhancing strategies should be focused on these groups of patients, and the most effective models of which should be evaluated in separate patient groups.

## Supporting Information

Appendix S1 **The Morisky Medication Adherence Scale (MMAS-8).**
(DOC)
