# Supplementary material for: Determinants of Medication Adherence to Antihypertensive Medications among a Chinese Population Using Morisky Medication Adherence Scale
Source: PLoS One. 2013 Apr 25;8(4):e62775. doi: 10.1371/journal.pone.0062775 (PMC3636185; doi:10.1371/journal.pone.0062775)
Supplement: Appendix S1 — The Morisky Medication Adherence Scale (MMAS-8). (DOC) [file pone.0062775.s001.doc]

**Appendix: The Morisky Medication Adherence Scale (MMAS-8)***

1). Do you sometimes forget to take your antihypertensive drugs?

2). People sometimes miss taking their antihypertensive medications for reasons other than forgetting. Thinking over the past two weeks, were there any days when you did not take your antihypertensive drugs?

3). Have you ever cut back or stopped taking medication without telling your doctor, because you felt worse when you took it?

4). When you travel or leave home, do you sometimes forget to bring along your antihypertensive medications?

5). Did you take your antihypertensive drugs yesterday?

6). When you feel like your hypertension is under control, do you sometimes stop taking your medicine?

7). Taking antihypertensive drugs every day is a real inconvenience for some people. Do you ever feel hassled about sticking to your blood pressure treatment plan?

8). How often do you have difficulty remembering to take your antihypertensive drugs?

* Use of the MMAS-8 is protected by US copyright laws. Permission for use is required. A license agreement is available from: Donald E. Morisky, ScD, ScM, MSPH, Professor, Department of Community Health Sciences, UCLA School of Public Health, 650 Charles E. Young Drive South, Los Angeles, CA 90095-1772.
